# The structure of the symptoms of major depression: exploratory and confirmatory factor analysis in depressed Han Chinese women

**DOI:** 10.1017/S003329171300192X

**Published:** 2013-08-07

**Authors:** Y. Li, S. Aggen, S. Shi, J. Gao, Y. Li, M. Tao, K. Zhang, X. Wang, C. Gao, L. Yang, Y. Liu, K. Li, J. Shi, G. Wang, L. Liu, J. Zhang, B. Du, G. Jiang, J. Shen, Z. Zhang, W. Liang, J. Sun, J. Hu, T. Liu, X. Wang, G. Miao, H. Meng, Y. Li, C. Hu, Y. Li, G. Huang, G. Li, B. Ha, H. Deng, Q. Mei, H. Zhong, S. Gao, H. Sang, Y. Zhang, X. Fang, F. Yu, D. Yang, T. Liu, Y. Chen, X. Hong, W. Wu, G. Chen, M. Cai, Y. Song, J. Pan, J. Dong, R. Pan, W. Zhang, Z. Shen, Z. Liu, D. Gu, X. Wang, X. Liu, Q. Zhang, J. Flint, K. S. Kendler

**Affiliations:** 1 Wellcome Trust Centre for Human Genetics, Oxford, UK; 2 Virginia Institute for Psychiatric and Behavioral Genetics, Department of Psychiatry, Virginia Commonwealth University, Richmond, VA, USA; 3 Shanghai Mental Health Center, Shanghai, P.R. China (PRC); 4 Huashan Hospital of Fudan University, Shanghai, PRC; 5 Chinese Traditional Hospital of Zhejiang, Hangzhou, Zhejiang, PRC; 6 No. 1 Hospital of Zhengzhou University, Zhengzhou, Henan, PRC; 7 Xinhua Hospital of Zhejiang Province, Hangzhou, Zhejiang, PRC; 8 No. 1 Hospital of Shanxi Medical University, Taiyuan, Shanxi, PRC; 9 ShengJing Hospital of China Medical University, Heping District, Shenyang, Liaoning, PRC; 10 No. 1 Hospital of Medical College of Xian Jiaotong University, Xian, Shaanxi, PRC; 11 Jilin Brain Hospital, Siping, Jilin, PRC; 12 The First Hospital of China Medical University, Heping District, Shenyang, Liaoning, PRC; 13 Mental Hospital of Jiangxi Province, Nanchang, Jiangxi, PRC; 14 Xian Mental Health Center, New Qujiang District, Xian, Shaanxi, PRC; 15 Beijing Anding Hospital of Capital University of Medical Sciences, Deshengmen wai, Xicheng District, Beijing, PRC; 16 Shandong Mental Health Center, Jinan, Shandong, PRC; 17 No. 3 Hospital of Sun Yat-sen University, Tianhe District, Guangzhou, Guangdong, PRC; 18 Hebei Mental Health Center, Baoding, Hebei, PRC; 19 Chongqing Mental Health Center, Jiangbei District, Chongqing, PRC; 20 Tianjin Anding Hospital, Hexi District, Tianjin, PRC; 21 No. 4 Hospital of Jiangsu University, Zhenjiang, Jiangsu, PRC; 22 Psychiatric Hospital of Henan Province, Xinxiang, Henan, PRC; 23 Nanjing Brain Hospital, Nanjing, Jiangsu, PRC; 24 Harbin Medical University, Nangang District, Haerbin, Heilongjiang, PRC; 25 Shenzhen Kang Ning Hospital, Luohu District, Shenzhen, Guangdong, PRC; 26 First Hospital of Hebei Medical University, Shijiazhuang, Hebei, PRC; 27 Guangzhou Brain Hospital (Guangzhou Psychiatric Hospital), Liwan District, Guangzhou, Guangdong, PRC; 28 No. 1 Hospital of Chongqing Medical University, Yuzhong District, Chongqing, PRC; 29 Dalian No. 7 Hospital, Ganjingzi District, Dalian, Liaoning, PRC; 30 No. 3 Hospital of Heilongjiang Province, Beian, Heilongjiang, PRC; 31 Wuhan Mental Health Center, Wuhan, Hubei, PRC; 32 Sichuan Mental Health Center, Mianyang, Sichuan, PRC; 33 Mental Health Institute of Jining Medical College, Dai Zhuang, Bei Jiao, Jining, Shandong, PRC; 34 Liaocheng No. 4 Hospital, Liaocheng, Shandong, PRC; 35 Mental Health Center of West China Hospital of Sichuan University, Wuhou District, Chengdu, Sichuan, PRC; 36 Suzhou Guangji Hospital, Suzhou, Jiangsu, PRC; 37 Anhui Mental Health Center, Hefei, Anhui, PRC; 38 Ningbo Kang Ning Hospital, Zhenhai District, Ningbo, Zhejiang, PRC; 39 Changchun Mental Hospital, Changchun, Jilin, PRC; 40 No. 2 Hospital of Lanzhou University, Lanzhou, Gansu, PRC; 41 Fuzhou Psychiatric Hospital, Cangshan District, Fuzhou, Fujian, PRC; 42 Harbin No. 1 Special Hospital, Haerbin, Heilongjiang, PRC; 43 Jining Psychiatric Hospital, North Dai Zhuang, Rencheng District, Jining, Shandong, PRC; 44 No. 2 Xiangya Hospital of Zhongnan University, Furong District, Changsha, Hunan, PRC; 45 Xijing Hospital of No. 4 Military Medical University, Xian, Shaanxi, PRC; 46 Mental Health Center of Shantou University, Shantou, Guangdong, PRC; 47 Tongji University Hospital, Shanghai, PRC; 48 Huaian No. 3 Hospital, Huaian, Jiangsu, PRC; 49 Huzhou No. 3 Hospital, Huzhou, Zhejiang, PRC; 50 Mudanjiang Psychiatric Hospital of Heilongjiang Province, Xinglong, Mudanjiang, Heilongjiang, PRC; 51 No. 1 Hospital of Jinan University, Guangzhou, Guangdong, PRC; 52 Qingdao Mental Health Center, Shibei District, Qingdao, Shandong, PRC; 53 Guangxi Longquanshan Hospital, Yufeng District, Liuzhou, PRC; 54 Daqing No. 3 Hospital of Heilongjiang Province, Ranghulu District, Daqing, Heilongjiang, PRC; 55 Tangshan No. 5 Hospital, Lunan District, Tangshan, Hebei, PRC; 56 Anshan Psychiatric Rehabilitation Hospital, Lishan District, Anshan, Liaoning, PRC; 57 Weihai Mental Health Center, ETDZ, Weihai, Shandong, PRC; 58 Renmin Hospital of Wuhan University, Wuchang District, Wuhan, Hubei, PRC; 59 Tianjin First Center Hospital, Hedong District, Tianjin, PRC; 60 Hainan Anning Hospital, Haikou, Hainan, PRC

**Keywords:** Atypical symptoms, China, cognitive symptoms, depression, factor analysis

## Abstract

**Background:**

The symptoms of major depression (MD) are clinically diverse. Do they form coherent factors that might clarify the underlying nature of this important psychiatric syndrome?

**Method:**

Symptoms at lifetime worst depressive episode were assessed at structured psychiatric interview in 6008 women of Han Chinese descent, age ⩾30 years with recurrent DSM-IV MD. Exploratory factor analysis (EFA) and confirmatoryfactor analysis (CFA) were performed in Mplus in random split-half samples.

**Results:**

The preliminary EFA results were consistently supported by the findings from CFA. Analyses of the nine DSM-IV MD symptomatic A criteria revealed two factors loading on: (i) general depressive symptoms; and (ii) guilt/suicidal ideation. Examining 14 disaggregated DSM-IV criteria revealed three factors reflecting: (i) weight/appetite disturbance; (ii) general depressive symptoms; and (iii) sleep disturbance. Using all symptoms (*n* = 27), we identified five factors that reflected: (i) weight/appetite symptoms; (ii) general retarded depressive symptoms; (iii) atypical vegetative symptoms; (iv) suicidality/hopelessness; and (v) symptoms of agitation and anxiety.

**Conclusions:**

MD is a clinically complex syndrome with several underlying correlated symptom dimensions. In addition to a general depressive symptom factor, a complete picture must include factors reflecting typical/atypical vegetative symptoms, cognitive symptoms (hopelessness/suicidal ideation), and an agitated symptom factor characterized by anxiety, guilt, helplessness and irritability. Prior cross-cultural studies, factor analyses of MD in Western populations and empirical findings in this sample showing risk factor profiles similar to those seen in Western populations suggest that our results are likely to be broadly representative of the human depressive syndrome.

## Introduction

Major depression (MD), forecast to become the second leading cause of disability worldwide by 2020 (Lopez & Murray, 1998), is characterized by diverse clinical symptoms (Lewis, 1934; APA, 1994), a subset of which became symptomatic criteria in the Diagnostic and Statistical Manual of Mental Disorders, fourth edition (DSM-IV) based on clinical observation and expert consensus (Kendler *et al.* 2010).

Whether the symptoms of MD can be well captured with one psychopathological dimension or require multiple dimensions has been long debated (Lewis, 1934; Grinker *et al.* 1961; MacFadyen, 1975). Recent analyses suggest that multiple factors are required to explain the co-occurrence of current depressive symptoms (e.g. Shafer, 2006; Romera *et al.* 2008; Bech *et al.* 2011; Van Loo *et al.* 2012). A twin study found three genetic factors underlying the DSM-IV A criteria for MD (Kendler *et al.* 2012).

The DSM-IV nine MD A diagnostic criteria evolved from the Feighner and Research Diagnostic Criteria (Feighner *et al.* 1972; Kendler *et al.* 2010), whose unidimensionality was later supported by empirical evidence from factor analysis using community samples (Muthén, 1989; Aggen *et al.* 2005). A meta-analysis of the factor structure for four commonly used depression questionnaires has reported two common factors including the general depression severity factor and a somatic symptom factor (Shafer, 2006). A recent review paper examined the results of both exploratory factor analysis (EFA) and confirmatory factor analysis (CFA) studies of nine DSM-IV criteria for MD (Van Loo *et al.* 2012). Results were diverse and did not provide conclusive evidence for any single typology of depressive symptoms. The authors noted that this literature was difficult to interpret because of differences in sample characteristics and specific items included in the factor analyses.

This study seeks to determine the factor structure of the symptoms of MD as reported for the lifetime worst episode in a very large sample of Han Chinese women with recurrent depression. Our sample size is sufficient to utilize EFA in a random split-half of the sample. The results are then verified using CFA in the second split-half.

We performed factor analyses on three sets of items: (i) the nine DSM-IV A criteria for MD (APA, 1994); (ii) 14 items representing the disaggregated DSM-IV criteria (i.e. increased and decreased appetite and weight, insomnia or hypersomnia, and psychomotor agitation or retardation); and (iii) 27 items, consisting of the 14 above mentioned plus 13 more detailed assessments of DSM criteria and symptoms of melancholia, anxiety and Beck's cognitive trio (Beck *et al.* 1980).

## Method

Subjects for this report came from the China, Oxford and VCU (Virginia Commonwealth University) Experimental Research on Genetic Epidemiology (CONVERGE) study (Flint *et al.* 2012). The analysis was based on the final phenotypic sample of 6008 cases recruited from 57 mental health centers and psychiatric departments of general hospitals in 45 cities in 23 provinces in China.

Subjects were females aged 30–60 years who reported two or more lifetime episodes of MD meeting DSM-IV criteria (APA, 1994). Their age at onset was between 14 and 55 years and each had four Han Chinese grandparents. Cases were excluded if they had a history of mania, psychosis outside of mood episodes or mental retardation, or had abused drugs or alcohol before their first depressive episode. The samples were primarily collected for a large genome-wide association study. Twin studies have suggested that the heritability of MD is higher in females and the genetic risk factors are not identical across sexes (Kendler *et al.* 2001, 2006). We therefore studied women only to reduce genetic heterogeneity.

Subjects were interviewed for an average of 2 h using a computerized assessment system that included assessment of psychopathology, demographic and personal characteristics, and psychosocial functioning. All interviewers, junior psychiatrists, postgraduate medical students, or senior nurses, were trained by the CONVERGE team for a minimum of 1 week. Interviews were recorded and a proportion of them edited for quality control. The study protocol was approved by the Ethical Review Board of Oxford University and the ethics committees in participating Chinese hospitals.

### Measures

The diagnosis of MD was established with the Composite International Diagnostic Interview (CIDI; World Health Organization lifetime version 2.1; Chinese version), which classifies diagnoses according to DSM-IV criteria (APA, 1994). The interview was originally translated into Mandarin by a team of psychiatrists in Shanghai Mental Health Center, with the translation reviewed and modified by members of the CONVERGE team. The interview was supplemented by sections from interviews used in the Virginia Adult Twin Study of Psychiatric and Substance Use Disorders (VATSPSUD) (Kendler & Prescott, 2006), and other items of interest to the investigators.

All interview sections, with built-in skip patterns, were computerized into a bilingual system of Mandarin and English that was installed on laptops. Once an interview was completed, a backup file containing the interview data was generated and, together with the audio record of the interview, uploaded to a server in Beijing and then transferred to Oxford. As operationalized in the CIDI, the symptoms analysed were based on those reported by the subject for the self-identified worst lifetime episode of MD.

### Statistical methods

The total sample was randomly divided into two halves. The first sample was used to perform an EFA and the second was used to perform a CFA for validating the EFA symptom structure. Factor analyses were performed using the Mplus program (Muthén & Muthén, 1998), with a weighted least squared means and variance adjusted (WLSMV) estimator that is designed for ordinal data (Flora & Curran, 2004). EFA was performed using a geomin oblique rotation. CFA model fit was evaluated using the Tucker–Lewis index (TLI; Tucker & Lewis, 1973), the comparative fit index (CFI; Bentler, 1990) and the root mean square error of approximation (RMSEA; Steiger, 1990). For the TLI and CFI, values between 0.90 and 0.95 are considered acceptable, and ⩾0.95 as good. For the RMSEA, good models have values ⩽0.05. To confirm that the split-sample procedure produced two random subsamples, we formally tested in Mplus for measurement invariance in our CFA data on the nine DSM-IV criteria. When we constrained factor loadings to be identical in the two subsamples, the robust *χ*^2^ difference, as expected, was not significant.

## Results

### EFA and CFA on nine MD DSM-IV A criteria

Endorsement frequencies for the nine MD DSM-IV A criteria for MD were uniformly high and similar in our two subsamples (Table 1). An EFA in sample 1 produced two eigenvalues exceeding unity. The first factor displayed higher loadings primarily on the somatic symptoms of depression and less prominently on mood symptoms, and reflected a ‘general depressive symptom’ factor. The second factor had highest loadings on suicidal ideation and worthlessness/guilt, and reflected a ‘cognitive symptoms’ factor (Table 1). The inter-factor correlation coefficient was  + 0.53. The best-fit CFA solution, two factors with all variable loadings ⩾0.30 from the EFA, had an excellent fit (RMSEA = 0.015, CFI = 0.98, TLI = 0.97), which was substantially better than those obtained with one factor (RMSEA = 0.031, CFI = 0.89, TLI = 0.86). The inter-factor correlation was  + 0.78. The loadings for the mood criteria A1 was stronger on the first factor and much weaker on the second factor in the CFA compared with the EFA. This factor, which still represented ‘general depressive symptoms’, had high loadings on seven of the nine A criteria. Factor 2, the ‘cognitive symptoms’ factor, had high loadings on the two criteria of worthlessness/guilt and suicidal ideation.

**Table 1.** Item percentage endorsement frequency on sample 1 and sample 2 for the nine DSM-IV criteria for major depression and loadings estimated from an EFA on sample 1 and a CFA on sample 2^a^

Items	EFA on sample 1			CFA on sample 2		
	Endorsement, %	Factor 1	Factor 2	Endorsement, %	Factor 1	Factor 2
Depressed mood	99.57	0.363	0.442	99.37	0.677	0.045
Anhedonia	98.87	0.266	0.206	98.84	0.542	
Significant weight or appetite disturbance	91.08	0.511	0.002	90.36	0.397	
Sleep disturbance	95.67	0.539	−0.054	95.58	0.353	
Psychomotor agitation or retardation	91.61	0.647	0.020	90.29	0.710	
Loss of energy or fatigue	93.53	0.719	−0.210	93.51	0.460	
Feeling of worthlessness	90.67	0.106	0.637	90.05		0.772
Diminished ability to think or concentrate, or indecisiveness	97.72	0.661	0.070	97.69	0.734	
Recurrent thoughts of death	76.32	−0.011	0.657	76.39		0.673

DSM-IV, Diagnostic and Statistical Manual of Mental Disorders, fourth edition; EFA, exploratory factor analysis; CFA, confirmatory factor analysis.

a Two-factor solution with loading cut-off at 0.3.

### EFA and CFA on the 14 disaggregated DSM-IV A criteria

The frequencies of item endorsement in the 14 disaggregated DSM-IV A criteria were similar in the two subsamples and more variable than with the nine criteria (Table 2). Atypical vegetative symptoms were endorsed by 7–13% of patients. The scree plot for the EFA analyses showed four factors with eigenvalues exceeding 1 and a clear ‘elbow’ at three factors. Furthermore, a solution with three factors was considerably more interpretable than one with four and resulted in factors reflecting ‘weight/appetite symptoms’, ‘general depressive symptoms’ and ‘sleep disturbance’ (Table 2) with modest inter-factor correlations (Table 3). In our CFA analyses (which produced the most interpretable results when including all EFA loadings ⩾0.30), the three-factor solution had better-fit indices (RMSEA = 0.032, CFI = 0.94, TLI = 0.93) than the four-factor solution (RMSEA = 0.035, CFI = 0.93, TLI = 0.91), validating our interpretation of the EFA. Loadings from the CFA were comparable with those found in the EFA, identifying three broadly similar factors. Inter-factor correlations were modest except between factors 1 and 3, which equaled −0.44 (Table 3).

**Table 2.** Item percentage endorsement frequency on sample 1 and sample 2 for the 14 disaggregated DSM-IV criteria for major depression and loadings estimated from an EFA on sample 1 and a CFA on sample 2^a^

Items	EFA on sample 1				CFA on sample 2			
	Endorsement, %	Factor 1	Factor 2	Factor 3	Endorsement, %	Factor 1	Factor 2	Factor 3
Depressed mood	99.57	0.093	0.684	−0.199	99.37		0.686	
Anhedonia	98.87	−0.082	0.408	0.130	98.84		0.478	
Loss of appetite	84.23	−0.722	0.629	0.000	82.83	0.674	0.643	
Loss of weight	60.15	−0.621	0.398	0.002	58.52	0.512	0.432	
Increase of appetite	9.43	0.738	0.010	0.366	9.74	−0.806		0.195
Increase of weight	7.11	0.842	−0.002	0.338	6.33	−0.879		0.223
Insomnia	92.99	−0.003	0.466	−0.596	91.87		0.345	−0.593
Hypersomnia	13.12	0.016	−0.004	0.765	13.53			0.947
Psychomotor retardation	76.69	−0.105	0.476	0.099	75.18		0.588	
Psychomotor agitation	72.19	−0.008	0.458	−0.134	72.91		0.448	
Loss of energy or fatigue	93.53	−0.100	0.500	−0.008	93.51		0.460	
Feeling of worthlessness	90.67	0.048	0.597	0.086	90.05		0.565	
Diminished ability to think or concentrate, or indecisiveness	97.72	0.015	0.723	−0.055	97.69		0.732	
Recurrent thoughts of death	76.32	−0.074	0.446	0.065	76.39		0.477	

DSM-IV, Diagnostic and Statistical Manual of Mental Disorders, fourth edition; EFA, exploratory factor analysis; CFA, confirmatory factor analysis.

a Items selected to load on CFA factors are based on EFA loading with a cut-off of 0.3.

**Table 3.** Inter-factor correlations for the EFA and CFA for the two-factor nine-item, three-factor 14-item and the five-factor 27-item factor analyses of the symptoms of major depression

EFA	F1	F2		CFA	F1	F2						
F1	1.000			F1	1.000							
F2	0.529	1.000		F2	0.777	1.000						
EFA	F1	F2	F3		CFA	F1	F2	F3				
F1	1.000				F1	1.000						
F2	0.209	1.000			F2	−0.115	1.000					
F3	0.182	0.099	1.000		F3	−0.443	0.106	1.000				
EFA	F1	F2	F3	F4	F5		CFA	F1	F2	F3	F4	F5
F1	1.000						F1	1.000				
F2	0.063	1.000					F2	0.513	1.000			
F3	−0.081	0.130	1.000				F3	−0.474	0.168	1.000		
F4	0.099	0.378	0.124	1.000			F4	0.337	0.513	0.136	1.000	
F5	−0.001	0.368	0.020	0.223	1.000		F5	0.394	0.870	0.176	0.619	1.000

EFA, Exploratory factor analysis; CFA, confirmatory factor analysis; F1, factor 1; F2, factor 2; F3, factor 3; F4, factor 4; F5, factor 5.

### EFA and CFA on all 27 individual symptoms assessed during worst depressive episode

Endorsement rates were variable for the 27 depression-related symptoms individually assessed during the worst lifetime episode and similar in our two subsamples (Table 4). More than 80% of our sample endorsed both key melancholic symptoms (e.g. lack of mood reactivity and distinct mood quality) and items assessing Beck's cognitive triad (e.g. hopelessness and worthlessness) indicating the clinical severity of the reported depressive episodes.

**Table 4.** Item percentage endorsement frequency on sample 1 and sample 2 for the 27 depressive symptoms and loadings estimated from an EFA on sample 1 and a CFA on sample 2^a^

Items	EFA on sample 1						CFA on sample 2					
	Endorsement, %	Factor 1	Factor 2	Factor 3	Factor 4	Factor 5	Endorsement, %	Factor 1	Factor 2	Factor 3	Factor 4	Factor 5
Depressed mood	99.57	−0.055	0.525	−0.187	0.129	0.161	99.37		0.703			
Anhedonia	98.87	0.025	0.437	0.049	0.117	−0.118	98.84		0.519			
Loss of appetite	84.23	0.710	0.398	−0.016	−0.021	−0.024	82.83	0.961	0.016			
Loss of weight	60.15	0.647	0.177	0.009	−0.043	0.111	58.52	0.636				
Increase of appetite	9.43	−0.653	−0.007	0.560	0.002	0.188	9.74	−0.169		0.800		
Increase of weight	7.11	−0.742	0.093	0.517	−0.053	−0.020	6.33	−0.144		0.902		
Insomnia	92.99	0.061	0.396	−0.489	−0.027	0.143	91.87		0.316	−0.440		
Hypersomnia	13.12	−0.091	−0.027	0.575	0.046	−0.011	13.53			0.503		
Psychomotor retardation	76.69	0.038	0.675	0.085	0.048	−0.281	75.18		0.962			−0.432
Psychomotor agitation	72.19	−0.033	0.334	−0.186	0.012	0.321	72.91		0.348			0.151
Loss of energy or fatigue	93.53	0.116	0.509	0.041	−0.122	0.044	93.51		0.439			
Worthlessness	82.39	−0.016	0.363	0.113	0.435	0.110	81.07		0.423		0.439	
Guilty or remorse	72.42	0.018	0.186	0.163	0.168	0.258	75.12					0.653
Trouble concentrating	92.15	−0.056	0.721	−0.043	−0.105	0.099	90.89		0.652			
Slower/mixed-up thoughts	89.30	−0.180	0.716	−0.008	0.016	−0.028	88.38		0.703			
Trouble making decision	83.36	−0.099	0.717	−0.005	0.031	−0.004	82.89		0.716			
Death thoughts	71.22	−0.010	−0.008	−0.072	0.963	−0.025	72.17				0.943	
Suicidal thoughts	61.72	−0.067	−0.011	−0.126	0.971	−0.010	60.92				0.946	
Loss of ability to enjoy good things	87.05	0.042	0.409	−0.002	0.217	−0.140	86.13		0.437		0.173	
Depression different from grief or loss	93.54	0.103	0.139	0.020	0.112	−0.122	92.46		0.271			
Worse mood in the morning	63.70	0.019	0.217	0.062	−0.072	0.079	60.65		0.290			
Loss of sexual drive	89.21	0.020	0.408	−0.127	0.042	−0.044	88.71		0.434			
Feel irritable or angry most of the time	74.59	−0.065	−0.021	−0.032	0.011	0.577	74.81					0.310
Hopeless	80.80	0.068	0.211	0.086	0.572	0.199	80.33		0.286		0.627	
Cry a lot	67.55	0.140	−0.089	0.143	0.168	0.368	67.34					0.337
Helpless	89.65	0.113	0.199	0.190	0.255	0.386	89.24				0.217	0.554
Feel nervous, jittery or anxious	89.58	−0.032	0.076	−0.086	−0.002	0.665	88.87					0.568

EFA, Exploratory factor analysis; CFA, confirmatory factor analysis.

a Items selected to load on CFA factors are based on EFA loading with a cut-off of 0.2.

The factor structure of these 27 items was initially examined by EFA (Table 3), resulting in eight eigenvalues exceeding 1 without a clear ‘elbow’ in the scree plot. With seven- and eight-factor solutions, estimated residual variances of some items became negative, indicating over-extraction. The five-factor EFA model was clinically more sensible than the six-factor solution. Similar to the results from the 14-item analysis, the first factor reflected ‘weight/appetite symptoms’ (Table 4). The second factor was also similar to that seen with the 14-item solution with a more prominent loading on psychomotor retardation. We called this the ‘general retarded depressive symptom’ factor. The third factor had highest loadings on the ‘atypical vegetative symptoms’ of increased appetite and weight, and hypersomnia. The fourth factor had prominent loadings on suicidal symptoms and Beck's triad of helplessness, hopelessness and worthlessness, and was called a ‘suicidal/hopeless’ factor. The fifth factor had prominent loadings on ‘agitated depressive symptoms’ including agitation, nervousness, guilt, irritability and crying. Inter-factor correlations were generally modest (Table 3), with the highest observed between the general depressive and suicidal/hopeless factors (+0.38).

In the CFA, when the number of factors equalled six or greater, standard errors of model parameter estimates as well as the factor scores could not be computed, which is also an indication of factor over-extraction. Both four-factor and five-factor solutions (with items of EFA loading cut-off of ⩾0.2) described the data well, with the five-factor solution resulting in slightly superior fits (RMSEA = 0.030, CFI = 0.953, TLI = 0.946 *v.* RMSEA = 0.030, CFI = 0.950, TLI = 0.943), again congruent with our clinical interpretation. The loadings closely resembled those found in the EFA. The general retarded depressive symptom factor had notable loadings (with absolute value ⩾0.2) on by far the most items (*n* = 15) with higher loadings for typical severe retarded depressive symptoms including several of the melancholic symptoms. The agitated depressive and suicidal/hopeless factors were next, with prominent loadings on six and five symptoms, respectively. The atypical vegetative and weight/appetite symptom factors were the smallest, with prominent loadings on four and two symptoms, respectively.

## Discussion

The goal of this study was to clarify the structure of the symptoms of MD experienced during the lifetime worst depressive episodes reported by Han Chinese women with recurrent DSM-IV MD (APA, 1994) ascertained in clinical settings throughout China. We conducted an EFA in a random split-half of the sample and then verified those results by CFA in the second random half. We conducted these analyses with: (i) the nine DSM-IV A criteria for MD; (ii) 14 items representing the disaggregated DSM-IV A criteria; and (iii) all 27 symptoms independently assessed in our interview for the worst lifetime depressive episode. Our preliminary EFA results on one half of our sample were consistently verified by the findings from CFA in the second random half.

Our study produced three major findings. First, we detected two factors underlying the nine DSM criteria for MD. The two ‘cognitive’ DSM criteria – worthlessness/guilt and suicidal ideation formed their own factor. Our results are convergent with prior analyses from the VATSPSUD (Lux & Kendler, 2010) that detected evidence for covert heterogeneity within the DSM-IV A criteria for MD, particularly between somatic and cognitive criteria (Lux & Kendler, 2010), as well as results from the Beck depression rating scale that cognitive symptoms form a factor independent of the somatic and affective features of depression (Beck *et al.* 1961). These findings suggest that in clinically depressed individuals, the DSM-IV A criteria for MD – which have changed very little since the days of the Research Diagnostic Criteria (Spitzer *et al.* 1975) and DSM-III (APA, 1980) – in fact reflect two inter-correlated but meaningfully distinct rather than one underlying psychopathological dimension.

It is particularly useful to compare our findings with those reported by Van Loo *et al.* (2012) who reviewed the results for nine studies of EFA and principal component analysis of DSM MD criteria. Of note, the sample size in our study is substantially greater than that of all nine of these prior studies. Contrary to the report of Aggen *et al.* (2005), all the studies reviewed detected at least two factors. Most commonly, as seen in our CFA, the core items of sad mood and loss of interest loaded on the first factor. However, the other features of the results of the individual studies were quite varied, further illustrating how dependent the results of factor analysis can be on the nature of the items examined and the sample studied.

Second, consistent with several prior multivariate analyses of MD criteria (Kendler *et al.* 1996; Sullivan *et al.* 1998; Matza *et al.* 2003) and depressive symptom scales (Bech *et al.* 2011), when atypical vegetative symptoms are well represented among the items, they form a distinct dimension of depressive symptomatology (Stewart *et al.* 1993). However, contrary to most prior studies of clinical criteria, perhaps because of our statistical power, we found that items reflecting sleep difficulty were moderately independent from the other vegetative symptoms reflecting changes in weight/appetite and formed their own factor. However, our results are consistent with a meta-analysis of the Hamilton Rating Scale for Depression that demonstrated separate factors in clinically depressed subjects for sleep problems and weight loss (Shafer, 2006). Sleep disturbances in severe MD may form an important psychopathological dimension at least partially independent of other neurovegetative changes (Kupfer, 1995).

Third, when we added to the DSM-IV disaggregated A criteria a range of other common depressive symptoms, a richer and potentially more informative picture emerged of the clinical complexity of severe depressive illness. We found replicated evidence for five depressive symptom factors. The largest factor contained prominent loadings on traditional mood changes, psychomotor retardation and several melancholic criteria, with modest loadings on certain cognitive symptoms. Items in this general retarded depressive symptom factor reflected six of the nine DSM IV A criteria: sad mood, anhedonia, psychomotor changes, worthlessness, fatigue, and concentration problems.

The next largest factor reflected agitated and anxious depressive symptoms, with additional high loadings on guilt, crying and feelings of helplessness. These symptoms resemble those describing the agitated depression (Koukopoulos & Koukopoulos, 1999) that was recognized as a subtype in the Research Diagnostic Criteria (Spitzer *et al.* 1975). These findings provide further evidence that anxiety symptoms can be a prominent part of the presentation of some forms of severe MD and may form a potentially important independent symptom factor as has been proposed for DSM-5 (First, 2011).

Perhaps the most interesting factor to emerge had prominent loadings on suicidal symptoms and Beck's triad of hopelessness, worthlessness and (somewhat more weakly) helplessness (Beck & Alford, 2008). Consistent with a large body of influential work (Beck *et al.* 1980; Beck & Alford, 2008), our results suggest that cognitive symptoms reflecting distorted views of the self, the world and the future form an important and largely independent dimension of depressive symptomatology which in this ascertained population is very closely related to suicidal ideation. These results also suggest that criterion A7 for MD does not reflect a unitary psychopathological construct, as its two major subcomponents – worthlessness and guilt – loaded on different depressive symptom factors.

Finally, similar to the analyses of the 14 items, the factor analyses of our 27 items identified two factors reflecting typical and atypical vegetative symptoms with weight/appetite and sleep items loading on separate factors.

Both our EFA and CFA utilized oblique rotations so that the depressive symptoms factors could inter-correlate. This approach produces a picture that, while more complex, is probably more realistic. For technical reasons (because CFA sets to zero low item loadings which partly capture inter-factor associations in an EFA), inter-factor correlations are typically higher in CFA than EFA. Because psychiatric symptoms are likely to be ‘messy’ and truly reflect more than one underlying dimension, the inter-factor correlations estimated with the more complex EFA factor loading patterns are probably more accurate than those produced by the stronger simple structure imposed in the CFAs. We would therefore emphasize only three of these correlations, all positive, as being especially noteworthy: between the general depressive and cognitive symptom factors in our nine-item analyses and, in our 27-item analyses between our general retarded depressive symptom factor and both the suicidal/hopeless and agitated depressive symptom factors.

Our results differ from two prior factor analyses of the nine DSM A criteria for MD in community samples (Muthén, 1989; Aggen *et al.* 2005), both of which detected only a single factor with substantially higher loadings on all criteria. Perhaps our ability to detect a second cognitive factor is related to our larger sample size and the greater clinical severity of our subjects. We are not aware of prior studies using similar methods and symptom content for our 14- and 27-item analyses. However, it is helpful to contextualize our findings by reviewing selectively earlier and more recent attempts to derive symptomatic factors from clinically depressed individuals. A classic factor analytic study of Grinker *et al.* (1961) of a wide variety of symptoms in 96 hospitalized depressed patients produced two factors similar to those we detected dominated by loadings on: (i) tension, anxiety and feeling jittery; and (ii) feeling hopeless, helpless, unworthy, and a failure (Grinker *et al.* 1961). The initial description of the Hamilton Rating Scale for Depression reported on 49 depressed patients four symptom factors, three of which resemble those we recovered including factors dominated by high loadings on: (i) suicide and guilt; (ii) agitation and anxiety; and (iii) insomnia and weight loss (Hamilton, 1960). Friedman, in another early study of 170 cases of psychotic depression using Grinker's 60-item depression rating scale, reported four symptom-dominated factors, three of which resembled factors that we isolated with high loadings on: (i) guilt and low self-esteem; (ii) appetite and sleep disturbance; and (iii) irritability, physical complaints and agitation (Friedman *et al.* 1963). An early analysis of the Beck Depression Scale on 254 hospitalized depressed patients yielded three interpretable factors of which two resembled those we had found with prominent loadings on: (i) guilt, self-accusation, sense of failure, self-punitive wishes and self-hate; and (ii) weight loss, loss of appetite, and sleep disturbance (Weckowicz *et al.* 1967).

Turning to more recent studies, in a meta-analysis of four popular self-report depression questionnaires, Shafer (2006) noted the consistency with which neurovegetative symptoms of depression loaded on an independent factor from other clinical symptoms. Bech *et al.* (2011), using principal component analysis applied to the 17-item Hamilton Rating Scale for Depression (HAMD_17_) scores from 4041 patients from the STAR*D (Sequenced Treatment Alternatives to Relieve Depression) study, found two factors, one reflecting general depressive severity and the second vegetative symptoms of weight, appetite and sleep disturbance. They also analysed the Inventory of Depressive Symptomatology (IDS-C_30_) in the same sample also reporting two factors. The first factor again included a broad array of depressive symptoms, while the second factor had notable loadings on panic, arousal, agitation, but also sleep difficulties (Parker, 2007). Finally, in 1049 primary care patients with MD, Romero *et al.* (2008) found four factors in the Zung Depression Scale, three of which were quite similar to factors we extracted with notable loadings on: (i) emptiness, hopelessness and suicidal rumination; (ii) agitation, irritability and crying spells; and (iii) decreased appetite and weight loss.

Our subjects were Han Chinese women with recurrent and severe MD recruited through clinical settings. We cannot be sure of the degree to which these results would extrapolate to men, to other ethnic groups, or to the milder forms of illness or symptom dimensions studied in community samples. However, the World Health Organization examined patients seeking care for depression in five international sites including East Asia and concluded that the clinical similarities of individuals far outweighed the modest cross-cultural differences (Sartorius *et al.* 1980). The factor structure of the Beck Depression Scale in Japan is very similar to that seen in Western populations (Kojima *et al.* 2002). Prior studies in this sample have shown that MD is associated, in a similar manner, with a range of risk factors previously demonstrated in Western samples including childhood sexual abuse (Cong *et al.* 2011), neuroticism (Xia *et al.* 2011), stressful life events (Tao *et al.* 2011) and low parental warmth (Gao *et al.* 2012), and has similar patterns of co-morbidity with anxiety disorders (Li *et al.* 2012) and dysthymia (Sang *et al.* 2011).

Epidemiological studies have found that rates of MD are lower in East Asia than in most other countries (Parker *et al.* 2001). There has been supporting evidence that the Chinese tend to deny depression or express it somatically (Kleinman, 1982; Parker *et al.* 2001). The leading theory is that this is a result of cultural stoicism and high levels of stigmatization. A recent epidemiological study in Taiwan supports this hypothesis. Individuals reporting MD were much more impaired than subjects reporting MD in parallel US studies and were only one-third as likely to seek professional help (Liao *et al.* 2012). This might also explain the clinical severity of our sample that was ascertained entirely through psychiatric treatment centers.

While it is possible that the patterns of depressive illness described in our sample are unique to this subgroup, this seems unlikely given prior cross-cultural research and the many parallels between our findings and previous empirical studies of MD in Western populations.

To our knowledge, this is the first study to examine the underlying symptom pattern of depression in the Han Chinese population using a carefully screened clinical sample of this size. The fact that we have identified common factors from Western samples provides evidence that there is more similarity than dissimilarities in depression in China and in Western countries. Future studies are needed to compare samples recruited using the same criteria from diverse ethnic groups to address more definitively the role played by culture and ethnicity in shaping the symptom dimensions of depression.

### Strengths and limitations

These results should be considered in the light of our potential methodological strengths and limitations. Four strengths are noteworthy. First, our phenotypic assessment was standardized and detailed with careful interview training and several quality-control features. Second, our sample was much larger than any previous similar effort and allowed us, with good power, to utilize EFA and CFA methods in split-half samples. Third, the goal of our sample collection strategy was to minimize heterogeneity that could add noise to our analysis. So, this sample was of a single sex with uniform ethnicity who suffered from severe recurrent depressive illness. Furthermore, drug and alcohol abuse were vanishingly rare in our sample of Han Chinese women, reducing a further major source of confound; also only 5% of them ever smoked. Fourth, the sample had a minimum age of 30 years and a mean age of 44.4 (s.d. = 8.9) years, reducing substantially the proportion that might eventually develop bipolar illness.

One major methodological limitation deserves comment. The results of factor analysis are critically dependent on the items analysed. We utilized a standardized interview for the DSM-IV criteria both in aggregated and disaggregated form. However, the other items added to form our final 27-item analysis reflected our own research interests. Furthermore, we could not include all the DSM-IV melancholic symptoms because some of them just assess the same symptoms as one of the A criteria at a more stringent level (e.g. melancholic criterion B4 is just a severe version of criterion A5). Including both items would cause estimation problems because the melancholic criterion would never be scored present in the absence of the relevant A criterion.
